# Supplementary material for: Mechanistic insights into ligand dissociation from the SARS-CoV-2 spike glycoprotein
Source: PLoS Comput Biol. 2024 Mar 7;20(3):e1011955. doi: 10.1371/journal.pcbi.1011955 (PMC10959368; doi:10.1371/journal.pcbi.1011955)
Supplement: S1 Text — (DOCX) [file pcbi.1011955.s003.docx]

**Text S1: Ligand Gaussian accelerated molecular dynamics (LiGaMD)**

LiGaMD builds upon the Gaussian accelerated molecular dynamics (GaMD) method^1–3^. GaMD is an enhanced sampling technique that works by adding a harmonic boost potential to smooth the biomolecular potential energy surface and reduce energy barriers. Details of the GaMD method have been described in previous studies^1–3^. The ligand GaMD (LiGaMD) method has been developed for more efficient sampling of protein-ligand binding^4^.

In the LiGaMD method, we consider a system composed of a ligand $L$ binding to a protein $P$ in a biological environment $E$. The system is comprised of $N$ atoms with positional coordinates $r\equiv\left\{ r_{1},\cdots,r_{N} \right\}$ and momenta $p\equiv\left\{ p_{1},\cdots,p_{N} \right\}$. The system Hamiltonian can be expressed as:

$H\left( r, p \right)=K\left( p \right)+V\left( r \right)$, (1)

where $K\left( p \right)$ and $V\left( r \right)$ represent the system’s kinetic and total potential energies, respectively. Next, we can break down the potential energy into the following terms:

$V\left( r \right)=V_{P,b}\left( r_{P} \right)+V_{L,b}\left( r_{L} \right)+V_{E,b}\left( r_{E} \right)$

$$+ V_{PP,nb}\left( r_{P} \right)+V_{LL,nb}\left( r_{L} \right)+V_{EE,nb}\left( r_{E} \right)$$

$+V_{PL,nb}\left( r_{PL} \right)+V_{PE,nb}\left( r_{PE} \right)+V_{LE,nb}\left( r_{LE} \right)$. (2)

where $V_{P,b}$, $V_{L,b}$ and $V_{E,b}$ are the bonded potential energies in protein $P$, ligand $L$ and environment $E$, respectively. $V_{PP,nb}$, $V_{LL,nb}$ and $V_{EE,nb}$ are the self nonbonded potential energies in protein $P$, ligand $L$ and environment $E$, respectively. $V_{PL,nb}$, $V_{PE,nb}$ and $V_{LE,nb}$ are the nonbonded interaction energies between $P\text{-}L$, $P\text{-}E$ and $L\text{-}E$, respectively. Classical molecular mechanics force fields calculate the nonbonded potential energies as^5–7^:

$V_{nb}=V_{elec}+V_{vdW}$. (3)

where $V_{elec}$ and $V_{vdW}$ denote the electrostatic and van der Waals potential energies, respectively. Note that ligand binding only involves the nonbonded interaction energies of the ligand, $V_{L,nb}\left( r \right)=V_{LL,nb}\left( r_{L} \right)+V_{PL,nb}\left( r_{PL} \right)+V_{LE,nb}\left( r_{LE} \right)$. In LiGaMD, we add a boost potential selectively to the ligand nonbonded potential energy:

$\Delta V_{L,nb}\left( r \right)=\left\{ \begin{aligned} \frac{1}{2}k_{L,nb}\left( E_{L,nb}-V_{L,nb}\left( r \right) \right)^{2}, &V_{L,nb}\left( r \right)<E_{L,nb} \\ 0, &V_{L,nb}\left( r \right)\geq E_{L,nb} \end{aligned} \right.$ (4)

where $E_{L,nb}$ is the threshold energy for applying boost potential and $k_{L,nb}$ is the harmonic constant. The LiGaMD simulation parameters are derived in the same manner as in the GaMD algorithm^3^. When $E$ is set to the lower bound as the system maximum potential energy ($E=V_{max}$), the effective harmonic force constant $k_{0}$ can be calculated as:

$k_{0}=\min\left( 1.0, k_{0}^{'} \right)=min(1.0, \frac{\sigma_{0}}{\sigma_{V}}\cdot\frac{V_{max}-V_{min}}{V_{max}-V_{avg}})$ (5)

where $V_{max}$, $V_{min}$, $V_{avg}$, and $\sigma_{V}$ are the maximum, minimum, average and standard deviation of the boosted system potential energy, and $\sigma_{0}$ is the user-specified upper limit of the standard deviation of $\Delta V$ (e.g., 10 $k_{B}T$) for proper reweighting. The harmonic constant is calculated as $k=k_{0}\cdot\frac{1}{V_{max}-V_{min}}$ with $0 \text{< }k_{0} \text{≤ }1$. Alternatively, when the threshold energy $E$ is set to its upper

bound $E=V_{min}+\frac{1}{k}$, $k_{0}$ is set to:

$k_{0}=k_{0}^{"}\equiv(1-\frac{\sigma_{0}}{\sigma_{V}})\frac{V_{max}-V_{min}}{V_{avg}-V_{min}}$ (6)

if $k_{0}$ is within the range of 0 to 1. Otherwise, $k_{0}$ is recalculated using Eq 5.

In addition to selectively boosting the bound ligand to accelerate its dissociation, an additional boost potential can be applied to unbound ligands, proteins, and solvent molecules, which can enhance the ligand rebinding to the protein. The second boost potential is determined using the system overall potential energy, excluding the nonbonded potential energy of the bound ligand:

$\Delta V_{D}\left( r \right)=\left\{ \begin{aligned} \frac{1}{2}k_{D}\left( E_{D}-V_{D}\left( r \right) \right)^{2}, &V_{D}\left( r \right)<E_{D} \\ 0, &V_{D}\left( r \right)\geq E_{D} \end{aligned} \right.$ (7)

where $E_{D}$ and $k_{D}$ are the corresponding threshold energy for applying the second boost potential and the harmonic constant, respectively. This leads to dual-boost LiGaMD with the total boost potential $\Delta V\left( r \right)=\Delta V_{L,nb}\left( r \right)+\Delta V_{D}(r)$. In this case, the strength of the individual boost potential term is determined by the user-defined threshold energies ($E_{L,nb}$ and $E_{D}$) and upper limits ($\sigma_{L,nb}$ and $\sigma_{D}$), the latter which determines the harmonic constants ($k_{L,nb}$ and $k_{D}$).

**References**

1. Wang, J. *et al.* Gaussian accelerated molecular dynamics: principles and applications. *WIREs Comput Mol Sci* **11**, e1521 (2021).

2. Miao, Y. & McCammon, J. A. Gaussian accelerated molecular dynamics: Theory, implementation, and applications. *Annu Rep Comput Chem* **13**, 231–278 (2017).

3. Miao, Y., Feher, V. A. & McCammon, J. A. Gaussian accelerated molecular dynamics: Unconstrained enhanced sampling and free energy calculation. *J Chem Theory Comput* **11**, 3584–3595 (2015).

4. Miao, Y., Bhattarai, A. & Wang, J. Ligand gaussian accelerated molecular dynamics (LiGaMD): characterization of ligand binding thermodynamics and kinetics. *J. Chem. Theory Comput.* **16**, 5526–5547 (2020).

5. Vanommeslaeghe, K. & MacKerell, A. D. CHARMM additive and polarizable force fields for biophysics and computer-aided drug design. *Biochimica et Biophysica Acta (BBA) - General Subjects* **1850**, 861–871 (2015).

6. Vanommeslaeghe, K. *et al.* CHARMM general force field: A force field for drug-like molecules compatible with the CHARMM all-atom additive biological force fields. *J. Comput. Chem.* **31**, 671–690 (2010).

7. Cornell, W. D. *et al.* A second generation force field for the simulation of proteins, nucleic acids, and organic molecules. *J Am Chem Soc* **118**, 2309 (1996).
