# Supplementary material for: Mechanistic insights into ligand dissociation from the SARS-CoV-2 spike glycoprotein
Source: PLoS Comput Biol. 2024 Mar 7;20(3):e1011955. doi: 10.1371/journal.pcbi.1011955 (PMC10959368; doi:10.1371/journal.pcbi.1011955)
Supplement: S2 Text — (DOCX) [file pcbi.1011955.s004.docx]

**Text S2: LiGaMD simulation input parameters**

An example of input parameters used in dual-boost LiGaMD simulations includes the following. The threshold energy is set to the upper bound for the ligand nonbonded boost potential and for the boost potential applied to the rest of the system. The user-specified upper limit of the boost potential is set to 6.0 kcal/mol for the ligand nonbonded term and 150.0 kcal/mol for the rest of the potential terms. The ligand nonbonded boost potential is applied to the ligand with residue number 3761.

Preparation Run:

***igamd = 7, irest_gamd = 0,***

***ntcmd = 2000000, nteb = 40000000, ntave = 500000,***

***ntcmdprep = 500000, ntebprep = 500000,***

***sigma0P = 6.0, sigma0D = 150.0, iEP = 2, iED = 2,***

***icfe = 1, ifsc = 1, gti_cpu_output = 0, gti_add_sc = 1,***

***timask1 = ':3761',***

***scmask1 = ':3761',***
