# Supplementary material for: Mechanistic insights into ligand dissociation from the SARS-CoV-2 spike glycoprotein
Source: PLoS Comput Biol. 2024 Mar 7;20(3):e1011955. doi: 10.1371/journal.pcbi.1011955 (PMC10959368; doi:10.1371/journal.pcbi.1011955)
Supplement: S1 Table — (DOCX) [file pcbi.1011955.s005.docx]

| **Chain** | **Site** | **Type** | **Structure** | **Sequence** |
| --- | --- | --- | --- | --- |
| A | N17 | FA2 | 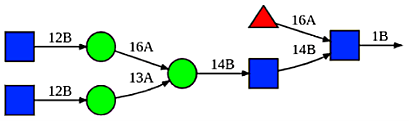 | bDGlcNAc(1→2)aDMan(1→6)[bDGlcNAc(1→2)aDMan(1→3)]  bDMan(1→4)bDGlcNAc(1→4)[aLFuc(1→6)]bDGlcNAc(1→)PROA-17 |
| A | N61 | M5 | 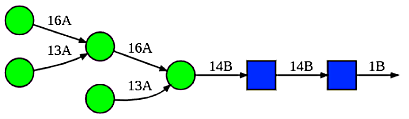 | aDMan(1→6)[aDMan(1→3)]aDMan(1→6) [aDMan(1→3)]bDMan(1→4)bDGlcNAc(1→4)bDGlcNAc(1→)PROA-61 |
| A | N122 | M5 | 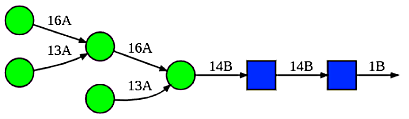 | aDMan(1→6)[aDMan(1→3)]aDMan(1→6) [aDMan(1→3)]bDMan(1→4)bDGlcNAc(1→4)bDGlcNAc(1→)PROA-122 |
| A | N165 | FA2G2S2 | 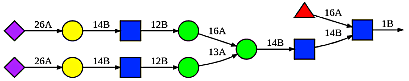 | xaDNeu5Ac(2→6)bDGal(1→4)bDGlcNAc(1→2)aDMan(1→6) [aDNeu5Ac(2→6)bDGal(1→4)bDGlcNAc(1→2)aDMan(1→3)]  bDMan(1→4)bDGlcNAc(1→4)[aLFuc(1→6)]bDGlcNAc(1→)PROA-165 |
| A | N234 | M8 | 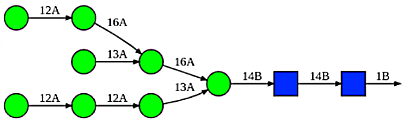 | aDMan(1→2)aDMan(1→6)[aDMan(1→3)]aDMan(1→6) [aDMan(1→2)aDMan(1→2)aDMan(1→3)]  bDMan(1→4)bDGlcNAc(1→4)bDGlcNAc(1→)PROA-234 |
| A | N282 | FA3 | 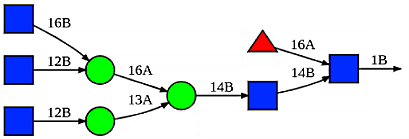 | bDGlcNAc(1→6)[bDGlcNAc(1→2)]aDMan(1→6)  [bDGlcNAc(1→2)aDMan(1→3)]bDMan(1→4)bDGlcNAc(1→4)  [aLFuc(1→6)]bDGlcNAc(1→)PROA-282 |
| A | N331 | FA2 | 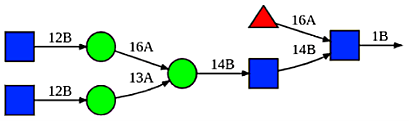 | bDGlcNAc(1→2)aDMan(1→6)[bDGlcNAc(1→2)aDMan(1→3)]  bDMan(1→4)bDGlcNAc(1→4)[aLFuc(1→6)]bDGlcNAc(1→)PROA-331 |
| A | N343 | FA2 | 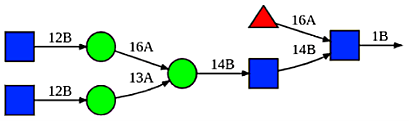 | bDGlcNAc(1→2)aDMan(1→6)[bDGlcNAc(1→2)aDMan(1→3)]  bDMan(1→4)bDGlcNAc(1→4)[aLFuc(1→6)]bDGlcNAc(1→)PROA-343 |
| A | N616 | A2 | 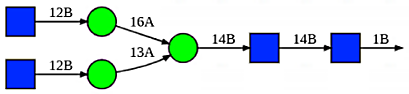 | bDGlcNAc(1→2)aDMan(1→6)[bDGlcNAc(1→2)aDMan(1→3)]  bDMan(1→4)bDGlcNAc(1→4)bDGlcNAc(1→)PROA-616 |
| A | N709 | M6 | 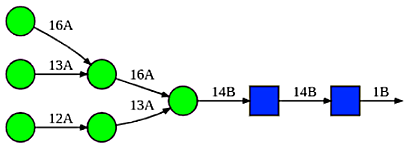 | aDMan(1→6)[aDMan(1→3)]aDMan(1→6)[aDMan(1→2)aDMan(1→3)]  bDMan(1→4)bDGlcNAc(1→4)bDGlcNAc(1→)PROA-709 |
| A | N717 | Hybrid G1 | 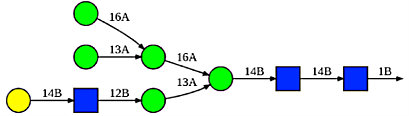 | bDGal(1→4)bDGlcNAc(1→2)aDMan(1→3)[aDMan(1→6)[aDMan(1→3)]  aDMan(1→6)]bDMan(1→4)bDGlcNAc(1→4)bDGlcNAc(1→)PROA-717 |
| A | N801 | M6 | 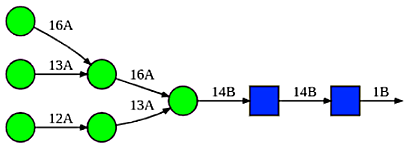 | aDMan(1→6)[aDMan(1→3)]aDMan(1→6)[aDMan(1→2)aDMan(1→3)]  bDMan(1→4)bDGlcNAc(1→4)bDGlcNAc(1→)PROA-801 |
| A | N1074 | FA2G2S1 | 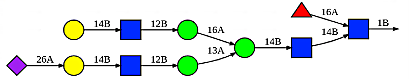 | aDNeu5Ac(2→6)bDGal(1→4)bDGlcNAc(1→2)aDMan(1→3) [bDGal(1→4)bDGlcNAc(1→2)aDMan(1→6)]bDMan(1→4)bDGlcNAc(1→4) [aLFuc(1→6)]bDGlcNAc(1→)PROA-1074 |
| A | N1098 | FA2 | 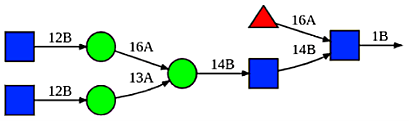 | bDGlcNAc(1→2)aDMan(1→6)[bDGlcNAc(1→2)aDMan(1→3)]  bDMan(1→4)bDGlcNAc(1→4)[aLFuc(1→6)]bDGlcNAc(1→)PROA-1098 |
| A | N1134 | FA1 | 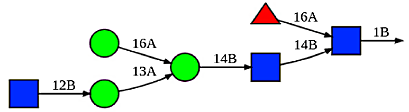 | bDGlcNAc(1→2)aDMan(1→3)[aDMan(1→6)]bDMan(1→4)bDGlcNAc(1→4) [aLFuc(1→6)]bDGlcNAc(1→)PROA-1134 |
| B | N17 | FA3 | 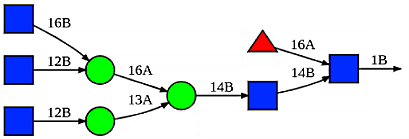 | bDGlcNAc(1→6)[bDGlcNAc(1→2)]aDMan(1→6)  [bDGlcNAc(1→2)aDMan(1→3)]bDMan(1→4)bDGlcNAc(1→4)  [aLFuc(1→6)]bDGlcNAc(1→)PROB-17 |
| B | N61 | M5 | 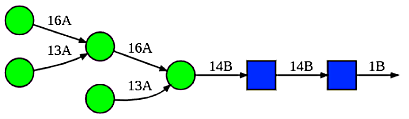 | aDMan(1→6)[aDMan(1→3)]aDMan(1→6)  [aDMan(1→3)]bDMan(1→4)bDGlcNAc(1→4)bDGlcNAc(1→)PROB-61 |
| B | N122 | FA2 | 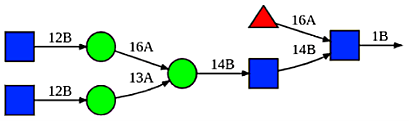 | bDGlcNAc(1→2)aDMan(1→6)[bDGlcNAc(1→2)aDMan(1→3)]  bDMan(1→4)bDGlcNAc(1→4)[aLFuc(1→6)]bDGlcNAc(1→)PROB-122 |
| B | N165 | M5 | 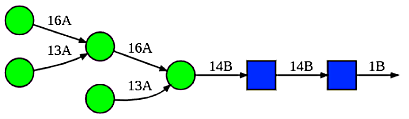 | aDMan(1→6)[aDMan(1→3)]aDMan(1→6)[aDMan(1→3)]  bDMan(1→4)bDGlcNAc(1→4)bDGlcNAc(1→)PROB-165 |
| B | N234 | M9 | 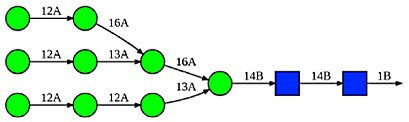 | aDMan(1→2)aDMan(1→6)[aDMan(1→2)aDMan(1→3)]aDMan(1→6)  [aDMan(1→2)aDMan(1→2)aDMan(1→3)]  bDMan(1→4)bDGlcNAc(1→4)bDGlcNAc(1→)PROB-234 |
| B | N282 | FA3G3S1 | 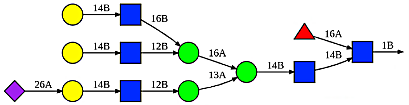 | aDNeu5Ac(2→6)bDGal(1→4)bDGlcNAc(1→2)aDMan(1→3)  [bDGal(1→4)bDGlcNAc(1→6)[bDGal(1→4)bDGlcNAc(1→2)]  aDMan(1→6)]bDMan(1→4)bDGlcNAc(1→4)  [aLFuc(1→6)]bDGlcNAc(1→)PROB-282 |
| B | N331 | FA2 | 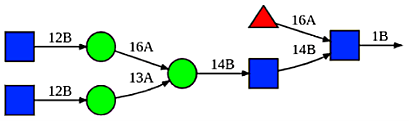 | bDGlcNAc(1→2)aDMan(1→6)[bDGlcNAc(1→2)aDMan(1→3)]  bDMan(1→4)bDGlcNAc(1→4)[aLFuc(1→6)]bDGlcNAc(1→)PROB-331 |
| B | N343 | FA1 | 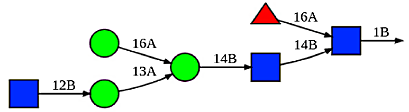 | bDGlcNAc(1→2)aDMan(1→3)[aDMan(1→6)]bDMan(1→4)bDGlcNAc(1→4)  [aLFuc(1→6)]bDGlcNAc(1→)PROB-343 |
| B | N616 | FA2 | 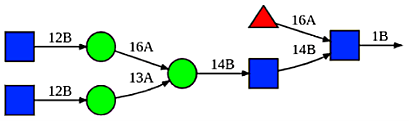 | bDGlcNAc(1→2)aDMan(1→6)[bDGlcNAc(1→2)aDMan(1→3)]  bDMan(1→4)bDGlcNAc(1→4)[aLFuc(1→6)]bDGlcNAc(1→)PROB-616 |
| B | N709 | M5 | 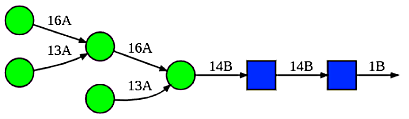 | aDMan(1→6)[aDMan(1→3)]aDMan(1→6)[aDMan(1→3)]  bDMan(1→4)bDGlcNAc(1→4)bDGlcNAc(1→)PROB-709 |
| B | N717 | M5 | 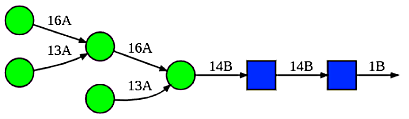 | aDMan(1→6)[aDMan(1→3)]aDMan(1→6)[aDMan(1→3)]  bDMan(1→4)bDGlcNAc(1→4)bDGlcNAc(1→)PROB-717 |
| B | N801 | M7 | 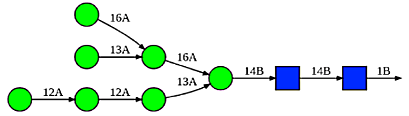 | aDMan(1→2)aDMan(1→2)aDMan(1→3)[aDMan(1→6)[aDMan(1→3)]  aDMan(1→6)]bDMan(1→4)bDGlcNAc(1→4)bDGlcNAc(1→)PROB-801 |
| B | N1074 | M5 | 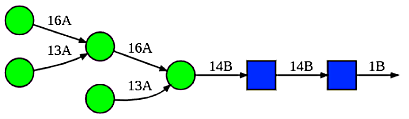 | aDMan(1→6)[aDMan(1→3)]aDMan(1→6)[aDMan(1→3)]  bDMan(1→4)bDGlcNAc(1→4)bDGlcNAc(1→)PROB-1074 |
| B | N1098 | A2 | 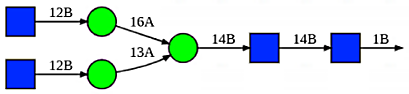 | bDGlcNAc(1→2)aDMan(1→6)[bDGlcNAc(1→2)aDMan(1→3)]  bDMan(1→4)bDGlcNAc(1→4)bDGlcNAc(1→)PROB-1098 |
| B | N1134 | FA3 | 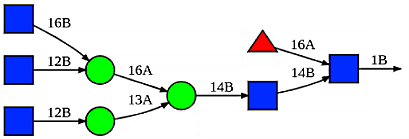 | bDGlcNAc(1→6)[bDGlcNAc(1→2)]aDMan(1→6)  [bDGlcNAc(1→2)aDMan(1→3)]bDMan(1→4)bDGlcNAc(1→4)  [aLFuc(1→6)]bDGlcNAc(1→)PROB-1134 |
| C | N17 | FA3 | 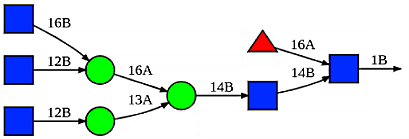 | bDGlcNAc(1→6)[bDGlcNAc(1→2)]aDMan(1→6)  [bDGlcNAc(1→2)aDMan(1→3)]bDMan(1→4)bDGlcNAc(1→4)  [aLFuc(1→6)]bDGlcNAc(1→)PROC-17 |
| C | N61 | M5 | 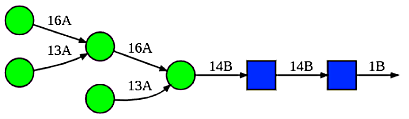 | aDMan(1→6)[aDMan(1→3)]aDMan(1→6) [aDMan(1→3)]  bDMan(1→4)bDGlcNAc(1→4)bDGlcNAc(1→)PROC-61 |
| C | N122 | M5 | 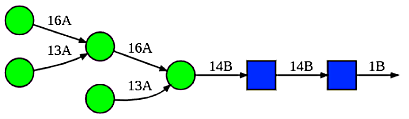 | aDMan(1→6)[aDMan(1→3)]aDMan(1→6) [aDMan(1→3)]  bDMan(1→4)bDGlcNAc(1→4)bDGlcNAc(1→)PROC-122 |
| C | N165 | FA2G2S1 | 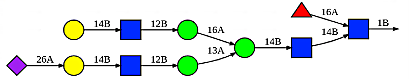 | aDNeu5Ac(2→6)bDGal(1→4)bDGlcNAc(1→2)aDMan(1→6)  [bDGal(1→4)bDGlcNAc(1→2)aDMan(1→3)]bDMan(1→4)bDGlcNAc(1→4) [aLFuc(1→6)]bDGlcNAc(1→)PROC-165 |
| C | N234 | M9 | 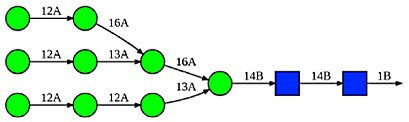 | aDMan(1→2)aDMan(1→6)[aDMan(1→2)aDMan(1→3)]aDMan(1→6)  [aDMan(1→2)aDMan(1→2)aDMan(1→3)]  bDMan(1→4)bDGlcNAc(1→4)bDGlcNAc(1→)PROC-234 |
| C | N282 | A2 | 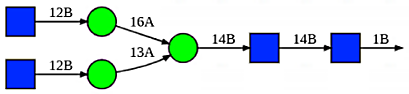 | bDGlcNAc(1→2)aDMan(1→6)[bDGlcNAc(1→2)aDMan(1→3)]  bDMan(1→4)bDGlcNAc(1→4)bDGlcNAc(1→)PROC-282 |
| C | N331 | FA3G3S1 | 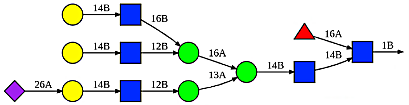 | aDNeu5Ac(2→6)bDGal(1→4)bDGlcNAc(1→2)aDMan(1→3)  [bDGal(1→4)bDGlcNAc(1→6)[bDGal(1→4)bDGlcNAc(1→2)]  aDMan(1→6)]bDMan(1→4)bDGlcNAc(1→4)  [aLFuc(1→6)]bDGlcNAc(1→)PROC-331 |
| C | N343 | FA2 | 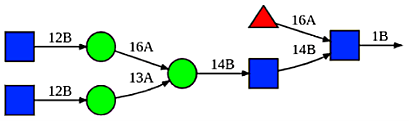 | bDGlcNAc(1→2)aDMan(1→6)[bDGlcNAc(1→2)aDMan(1→3)]  bDMan(1→4)bDGlcNAc(1→4) [aLFuc(1→6)]bDGlcNAc(1→)PROC-343 |
| C | N616 | FA2 | 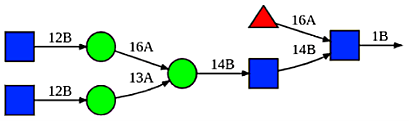 | bDGlcNAc(1→2)aDMan(1→6)[bDGlcNAc(1→2)aDMan(1→3)]  bDMan(1→4)bDGlcNAc(1→4)[aLFuc(1→6)]bDGlcNAc(1→)PROC-616 |
| C | N709 | M5 | 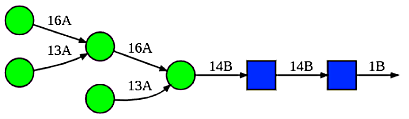 | aDMan(1→6)[aDMan(1→3)]aDMan(1→6) [aDMan(1→3)]  bDMan(1→4)bDGlcNAc(1→4)bDGlcNAc(1→)PROC-709 |
| C | N717 | M6 | 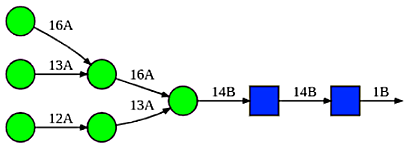 | aDMan(1→6)[aDMan(1→3)]aDMan(1→6)[aDMan(1→2)aDMan(1→3)]  bDMan(1→4)bDGlcNAc(1→4)bDGlcNAc(1→)PROC-717 |
| C | N801 | M5 | 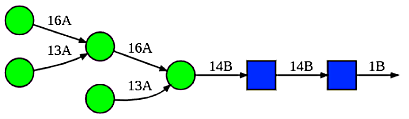 | aDMan(1→6)[aDMan(1→3)]aDMan(1→6) [aDMan(1→3)]  bDMan(1→4)bDGlcNAc(1→4)bDGlcNAc(1→)PROC-801 |
| C | N1074 | M5 | 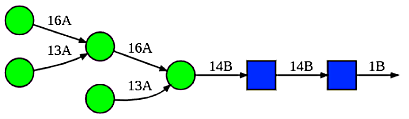 | aDMan(1→6)[aDMan(1→3)]aDMan(1→6) [aDMan(1→3)]  bDMan(1→4)bDGlcNAc(1→4)bDGlcNAc(1→)PROC-1074 |
| C | N1098 | Hybrid G1S1 | 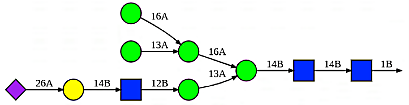 | aDNeu5Ac(2→6)bDGal(1→4)bDGlcNAc(1→2)aDMan(1→3)  [aDMan(1→6)[aDMan(1→3)]aDMan(1→6)]  bDMan(1→4)bDGlcNAc(1→4)bDGlcNAc(1→)PROC-1098 |
| C | N1134 | FA2 | 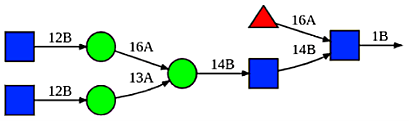 | bDGlcNAc(1→2)aDMan(1→6)[bDGlcNAc(1→2)aDMan(1→3)]  bDMan(1→4)bDGlcNAc(1→4)[aLFuc(1→6)]bDGlcNAc(1→)PROC-1134 |
