# Supplementary material for: Mechanistic insights into ligand dissociation from the SARS-CoV-2 spike glycoprotein
Source: PLoS Comput Biol. 2024 Mar 7;20(3):e1011955. doi: 10.1371/journal.pcbi.1011955 (PMC10959368; doi:10.1371/journal.pcbi.1011955)
Supplement: S2 Table — This table outlines the detailed steps involved in system preparation and LiGaMD simulation for spike glycoprotein systems. The minimization processes were carried out in stages including the minimization of protein hydrogen atoms, protein backbone, protein, ligand, and glycans. Followed by the addition of a water box, minimization of water molecules, and then minimization of the entire system. After executing water equilibration, the systems were gradually equilibrated at 50, 100, 150, 200, 250 and 310 K. LiGaMD preparation was performed by first performing conventional MD to collect the boost parameters, then applying a fixed boost potential, and then adjusting the boost potential while updating the boost parameters. The LiGaMD production simulation is run with the final updated boost parameters for either 500 ns or until successful ligand dissociation. (DOCX) [file pcbi.1011955.s006.docx]

| **Simulation protocol** | **Stage** | **Number of steps** |
| --- | --- | --- |
| System minimization | Minimization of hydrogen | 5,000 steps |
|  | Minimization of backbone | 25,000 steps |
|  | Minimization of protein, glycans, and ligands | 25,000 steps |
|  | Minimization of water molecules | 5,000 steps |
|  | Minimization of entire system | 25,000 steps |
| System equilibrium | Equilibration of water molecules | 200 ps |
|  | Equilibration of entire system at 50, 100, 150, 200, and 250 K | 100 ps (20 ps at each temperature) |
|  | cMD simulation at 310 K | 20.2 ns |
| LiGaMD preparation | cMD simulation | 4 ns |
|  | LiGaMD with fixed boost parameters | 1 ns |
|  | LiGaMD with updated boost parameters | 79 ns |
| LiGaMD production | LiGaMD simulation | 500 ns or until dissociation |
