# Supplementary material for: Mechanistic insights into ligand dissociation from the SARS-CoV-2 spike glycoprotein
Source: PLoS Comput Biol. 2024 Mar 7;20(3):e1011955. doi: 10.1371/journal.pcbi.1011955 (PMC10959368; doi:10.1371/journal.pcbi.1011955)
Supplement: S3 Table — The number of water molecules within distances of 5, 6, 7, and 8 Å around LA at various stages along dissociation pathways A and B for model systems LA‐RBDACC and LA‐RBDABCC. Please refer to S2 Fig for each dissociation state. (DOCX) [file pcbi.1011955.s007.docx]

| System | Time | Reference Figure | Dissociation State | Number of Water Contacts | | | |
| --- | --- | --- | --- | --- | --- | --- | --- |
|  |  |  |  | ≤ 5 Å | ≤ 6 Å | ≤ 7 Å | ≤ 8 Å |
| $\text{LA-RBD}_{\text{ABC}}^{C}$ | 0 ns | Fig S2A | Fully Bound | 0 | 0 | 0 | 0 |
| $\text{LA-RBD}_{\text{AC}}^{C}$ | 0 ns | Fig S2A | Fully Bound | 0 | 0 | 0 | 0 |
| $\text{LA-RBD}_{\text{ABC}}^{C}$ | 40 ns | Fig S2B | Flipping toward Path A | 0 | 1 | 2 | 2 |
| $\text{LA-RBD}_{\text{AC}}^{C}$ | 26 ns | Fig S2B | Flipping toward Path B | 0 | 1 | 1 | 1 |
| $\text{LA-RBD}_{\text{ABC}}^{C}$ | 224 ns | Fig S2C | Moving along Path A | 6 | 11 | 19 | 25 |
| $\text{LA-RBD}_{\text{AC}}^{C}$ | 182 ns | Fig S2D | Moving along Path B | 6 | 10 | 15 | 25 |
| $\text{LA-RBD}_{\text{ABC}}^{C}$ | 344 ns | Fig S2E | Fully dissociated from Path A | 37 | 68 | 110 | 154 |
| $\text{LA-RBD}_{\text{ABC}}^{C}$ | 288 ns | Fig S2F | Fully dissociated from Path B | 22 | 37 | 67 | 99 |
