# Supplementary material for: Mechanistic insights into ligand dissociation from the SARS-CoV-2 spike glycoprotein
Source: PLoS Comput Biol. 2024 Mar 7;20(3):e1011955. doi: 10.1371/journal.pcbi.1011955 (PMC10959368; doi:10.1371/journal.pcbi.1011955)
Supplement: S4 Table — The Cα distance between E340 and A372 was measured. The RBDs that cannot be determined either up or down conformation are noted as N/A. (DOCX) [file pcbi.1011955.s008.docx]

| **PDB ID** | **Receptor-binding Domain** | **Ligand** | **Conformation** | **E340-A372 Distance** |
| --- | --- | --- | --- | --- |
| 6ZB5^1^ | RBD_A_ | Linoleate | Down | 13.31 |
|  | RBD_B_ | Linoleate | Down | 13.31 |
|  | RBD_C_ | Linoleate | Down | 13.31 |
| 6VXX^2^ | RBD_A_ | N/A | Down | 18.21 |
|  | RBD_B_ | N/A | Down | 18.21 |
|  | RBD_C_ | N/A | Down | 18.21 |
| 6VSB^3^ | RBD_A_ | N/A | Up | 18.27 |
|  | RBD_B_ | N/A | Down | 18.25 |
|  | RBD_C_ | N/A | Down | 17.14 |
| 6VYB^2^ | RBD_A_ | N/A | Down | 18.00 |
|  | RBD_B_ | N/A | Up | 18.38 |
|  | RBD_C_ | N/A | Down | 18.03 |
| 6ZGG^4^ | RBD_A_ | N/A | Down | 13.57 |
|  | RBD_B_ | N/A | Up | 11.93 |
|  | RBD_C_ | N/A | Down | 12.10 |
| 6ZGH^4^ | RBD_A_ | N/A | Down | 11.69 |
|  | RBD_B_ | N/A | Down | 12.43 |
| 7CAB^5^ | RBD_A_ | N/A | Down | 14.91 |
|  | RBD_B_ | N/A | Down | 14.91 |
|  | RBD_C_ | N/A | Down | 14.91 |
| 7DK3^6^ | RBD_A_ | N/A | Down | 16.67 |
|  | RBD_B_ | N/A | Down | 17.33 |
|  | RBD_C_ | N/A | Up | 15.20 |
| 7WZ1^7^ | RBD_A_ | N/A | Down | 15.55 |
|  | RBD_B_ | N/A | Up | 12.4 |
|  | RBD_C_ | N/A | Down | 17.72 |
| 7WZ2^7^ | RBD_A_ | N/A | Down | 18.97 |
|  | RBD_B_ | N/A | Up | 17.79 |
|  | RBD_C_ | N/A | Down | 18.63 |
| 6VW1^8^ | RBD_A_ | N/A | N/A | 19.37 |
|  | RBD_B_ | N/A | N/A | 19.20 |
| 7E3J^9^ | RBD_A_ | N/A | N/A | 19.75 |
| 7U0N^10^ | RBD_A_ | N/A | N/A | 19.27 |
|  | RBD_B_ | ACE2 | N/A | 19.10 |
| 7W8S^11^ | RBD_A_ | ACE2 | N/A | 18.81 |
| 7WA1^11^ | RBD_A_ | ACE2 | N/A | 19.00 |
| 7C8D^12^ | RBD_A_ | ACE2 | N/A | 19.38 |
| 7C8J^13^ | RBD_A_ | ACE2 | N/A | 18.64 |
| 7CAH^5^ | RBD_A_ | H014 Fab | N/A | 17.29 |
| 7LM8^14^ | RBD_A_ | CV38-142 and COVA1-16 Fabs | N/A | 18.83 |
| 7LM9^14^ | RBD_A_ | CV38-142 Fab | N/A | 19.37 |
| 7TB8^15^ | RBD_A_ | A19-61.1 antibody | Up | 15.69 |
|  | RBD_B_ | A19-61.1 antibody | Down | 16.43 |
|  | RBD_C_ | B1-182.1 antibody | Up | 15.89 |
| 7TBF^15^ | RBD_A_ | B1-182.1 and A19-61.1 antibodies | N/A | 15.48 |
| 7WCD^16^ | RBD_A_ | TAU-2212 antibody | Down | 17.45 |
|  | RBD_B_ | TAU-2212 antibody | Down | 17.44 |
|  | RBD_C_ | TAU-2212 antibody | Down | 17.45 |
| 7WOG^7^ | RBD_A_ | 553-49 antibody | N/A | 16.59 |
| 7X7O^17^ | RBD_A_ | UT28K Fab | N/A | 14.95 |
| 8C8P^18^ | RBD_A_ | 10D12 antibody | N/A | 19.07 |

**References**

1. Toelzer, C. *et al.* Free fatty acid binding pocket in the locked structure of SARS-CoV-2 spike protein. *Science* **370**, 725–730 (2020).

2. Walls, A. C. *et al.* Structure, function, and antigenicity of the SARS-CoV-2 spike glycoprotein. *Cell* **181**, 281–292 (2020).

3. Wrapp, D. *et al.* Cryo-EM structure of the 2019-nCoV spike in the prefusion conformation. *Science* **367**, 1260–1263 (2020).

4. Wrobel, A. G. *et al.* SARS-CoV-2 and bat RaTG13 spike glycoprotein structures inform on virus evolution and furin-cleavage effects. *Nat Struct Mol Biol* **27**, 763–767 (2020).

5. Lv, Z. *et al.* Structural basis for neutralization of SARS-CoV-2 and SARS-CoV by a potent therapeutic antibody. (2020).

6. Conformational dynamics of SARS-CoV-2 trimeric spike glycoprotein in complex with receptor ACE2 revealed by cryo-EM. *Sci Adv* **7**, eabe5575 (2021).

7. Zhan, W. *et al.* Structural study of SARS-CoV-2 antibodies identifies a broad-spectrum antibody that neutralizes the omicron variant by disassembling the spike trimer. *J Virol* **96**, e00480-22 (2022).

8. Shang, J. *et al.* Structural basis of receptor recognition by SARS-CoV-2. *Nature* **581**, 221–224 (2020).

9. Zhang, Z. *et al.* The molecular basis for SARS-CoV-2 binding to dog ACE2. *Nat Commun* **12**, 4195 (2021).

10. Geng, Q. *et al.* Structural basis for human receptor recognition by SARS-CoV-2 omicron variant BA.1. *J Virol* **96**, e00249-22 (2022).

11. Su, C. *et al.* Molecular basis of mink ACE2 binding to SARS-CoV-2 and its mink-derived variants. *J Virol* **96**, e00814-22 (2022).

12. Wu, L. *et al.* Broad host range of SARS-CoV-2 and the molecular basis for SARS-CoV-2 binding to cat ACE2. *Cell Discov* **6**, 68 (2020).

13. Liu, K. *et al.* Cross-species recognition of SARS-CoV-2 to bat ACE2. *Proc. Natl. Acad. Sci. U.S.A.* **118**, e2020216118 (2021).

14. Liu, H. *et al.* A combination of cross-neutralizing antibodies synergizes to prevent SARS-CoV-2 and SARS-CoV pseudovirus infection. *Cell Host & Microbe* **29**, 806-818.e6 (2021).

15. Zhou, T. *et al.* Structural basis for potent antibody neutralization of SARS-CoV-2 variants including B.1.1.529. *Science* **376**, eabn8897 (2022).

16. Li, R. *et al.* Conformational flexibility in neutralization of SARS-CoV-2 by naturally elicited anti-SARS-CoV-2 antibodies. *Commun Biol* **5**, 789 (2022).

17. Ozawa, T. *et al.* Novel super-neutralizing antibody UT28K is capable of protecting against infection from a wide variety of SARS-CoV-2 variants. *mAbs* **14**, 2072455 (2022).

18. Du, W. *et al.* Avidity engineering of human heavy-chain-only antibodies mitigates neutralization resistance of SARS-CoV-2 variants. *Front. Immunol.* **14**, 1111385 (2023).
