# Supplementary figures and images for: Mechanistic insights into ligand dissociation from the SARS-CoV-2 spike glycoprotein
Source: PLoS Comput Biol. 2024 Mar 7;20(3):e1011955. doi: 10.1371/journal.pcbi.1011955 (PMC10959368; doi:10.1371/journal.pcbi.1011955)

A

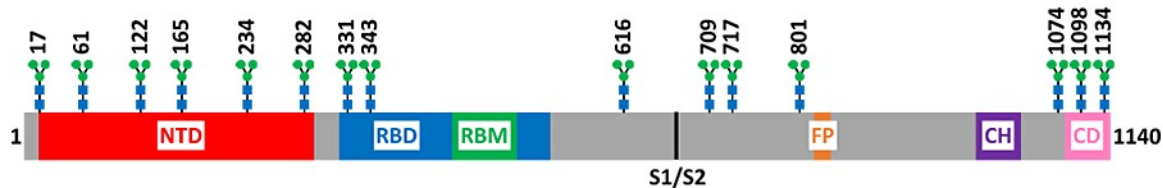

B

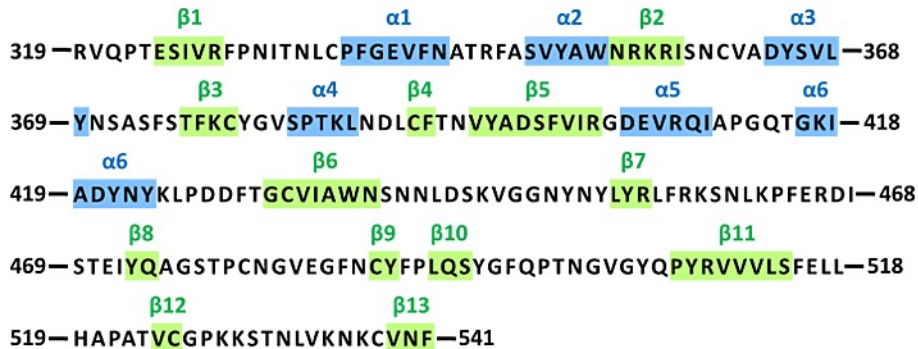

Supplement: S1 Fig — (A) Schematic of the spike protein primary structure for one chain: N-terminal domain (NTD, 16−291), receptor-binding domain (RBD, 319−541), receptor-binding motif (RBM, 438–506), furin cleavage site (S1/S2), fusion peptide (FP, 817−834), central helix (CH, 987−1034), connecting domain (CD, 1080−1140). Representative icons indicate N-glycans (blue and green) at N17, N61, N122, N165, N234, N282, N331, N343, N616, N709, N717, N801, N1074, N1098, and N1134. (B) Sequence and secondary structures of the spike RBD. Blue and green indicate α helices and β sheets, respectively. (PDF) [file pcbi.1011955.s009.pdf]

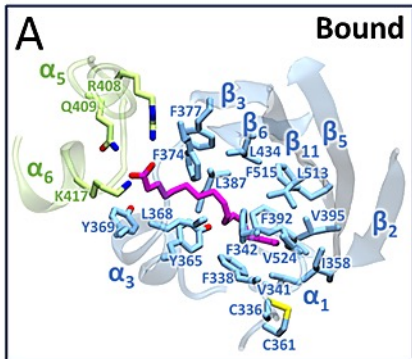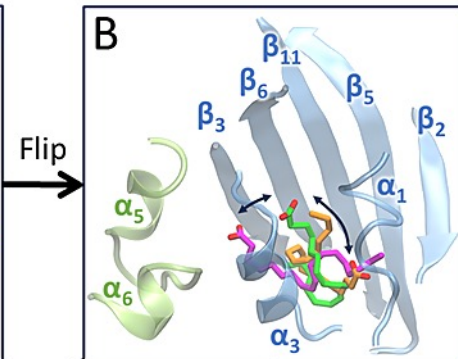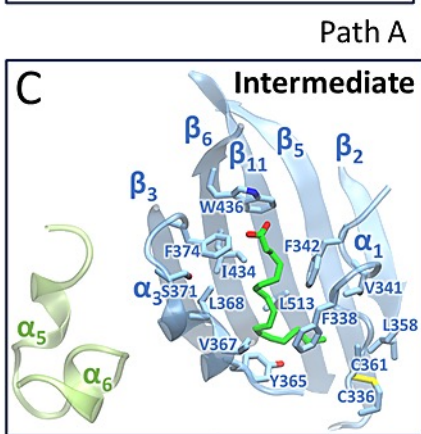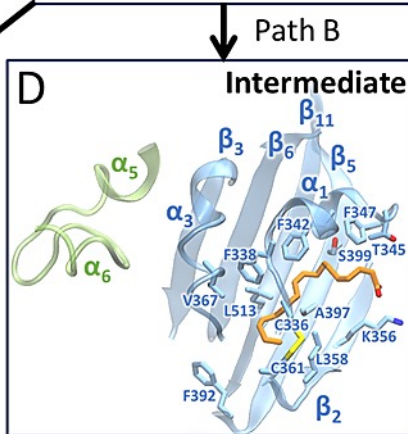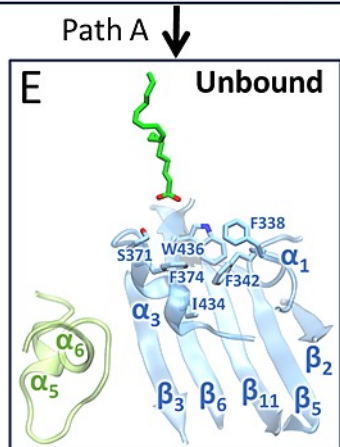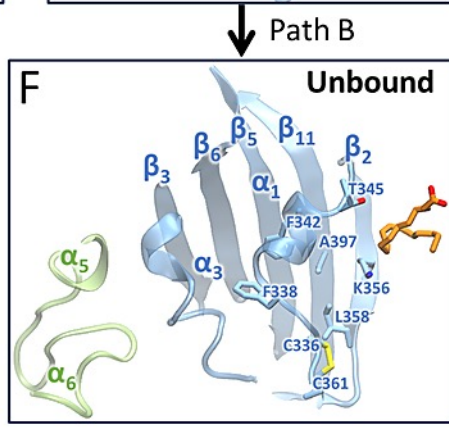

Supplement: S2 Fig — (A) LA is tightly bound in the FFA binding pocket. (B) LA undergoes a flipping motion, initiating the dissociation process. (C) LA moves along Path A. (D) LA moves along Path B. (E) LA fully dissociates from the binding pocket along Path A. (F) LA fully dissociates from the binding pocket along Path B. (PDF) [file pcbi.1011955.s010.pdf]

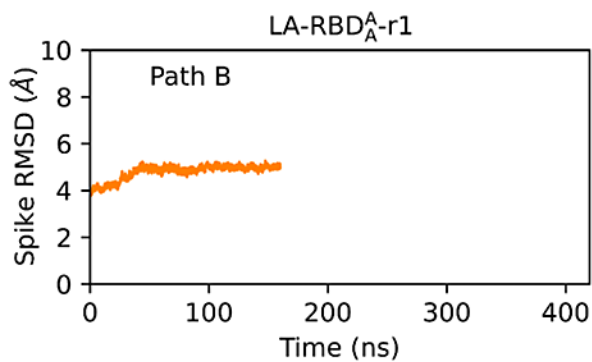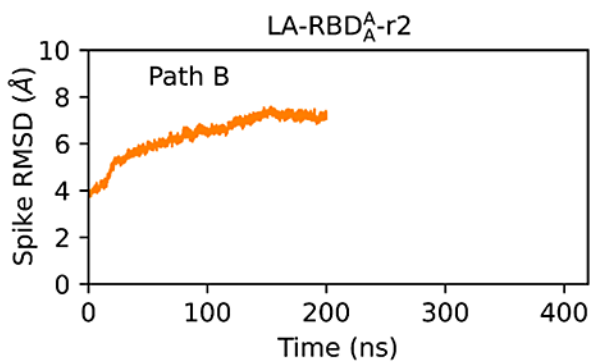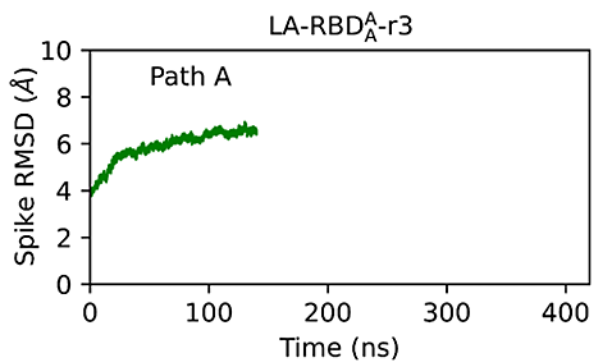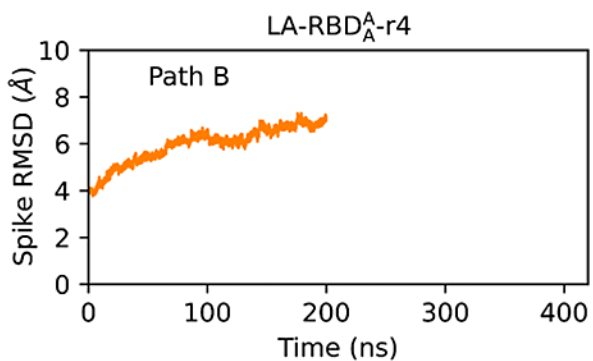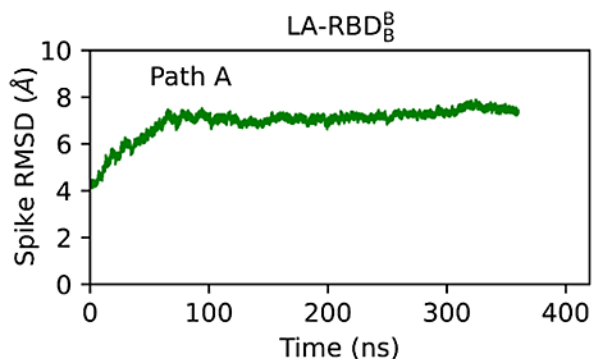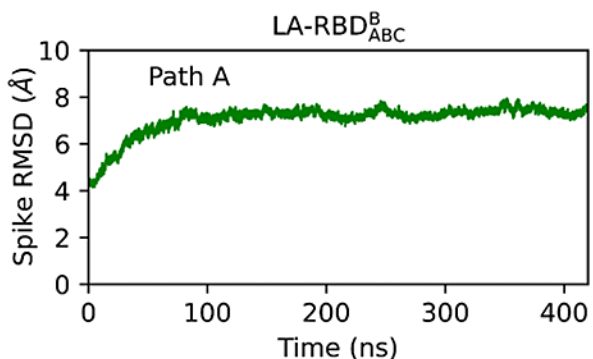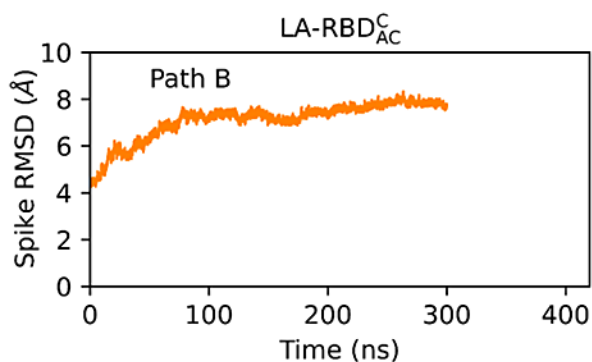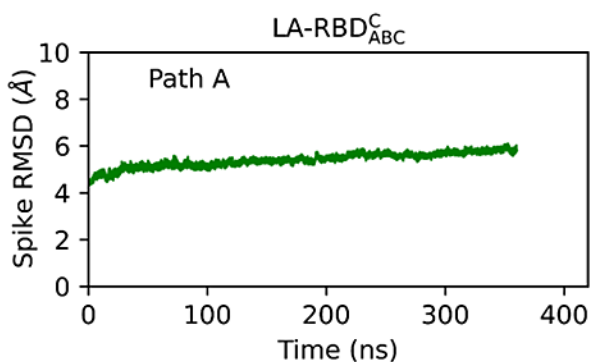

Supplement: S3 Fig — Green and orange indicate trajectories dissociating along Path A and B, respectively. (PDF) [file pcbi.1011955.s011.pdf]

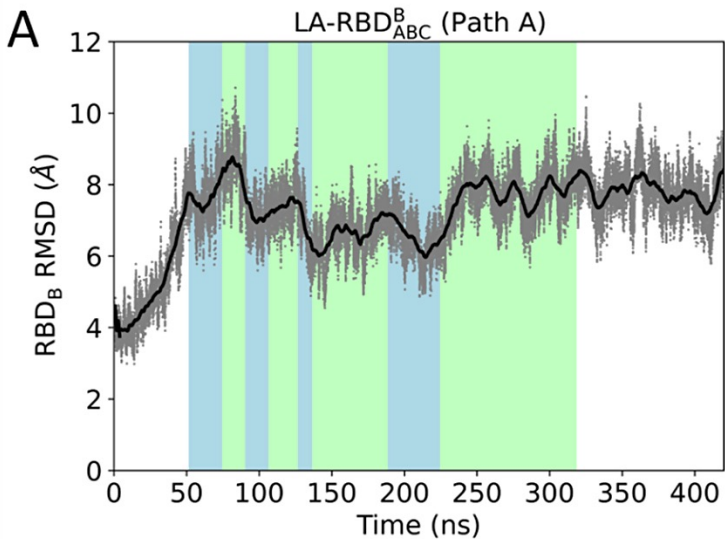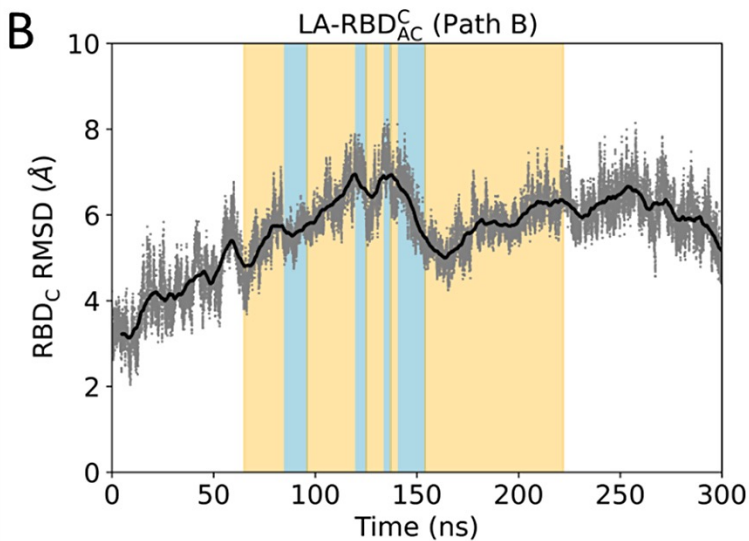

Supplement: S4 Fig — RMSD analysis of the spike RBD in complex with a boosted LA for trajectories, LA‐RBDABCB (A) and LA‐RBDACA (B). The color highlights different LA states: fully bound (blue), traveling along Path A (green), and moving along Path B (orange). (PDF) [file pcbi.1011955.s012.pdf]

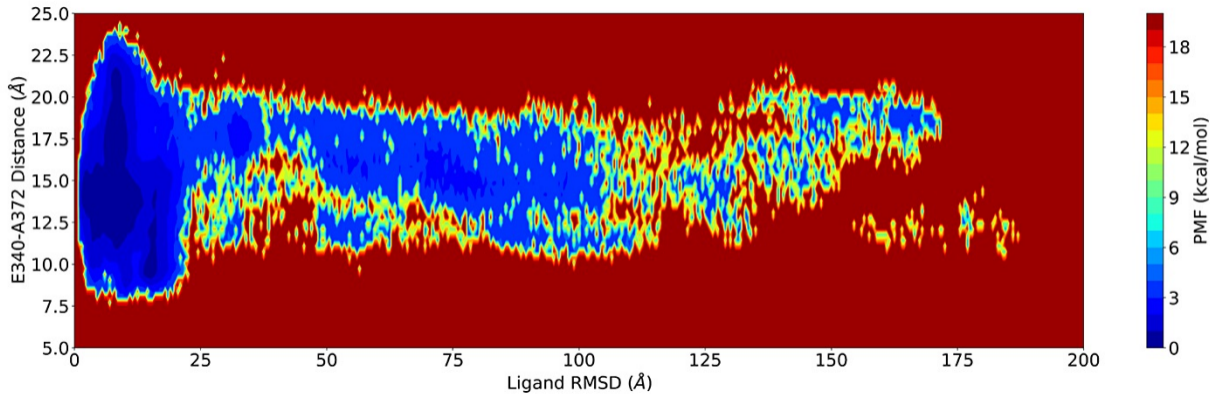

Supplement: S7 Fig — The plot depicts the PMF changes with the ligand RMSD and the gate distance between E340 and A372, including the states after LA fully dissociates with the ligand RMSD exceeds 30 Å. (PDF) [file pcbi.1011955.s015.pdf]

**A****Path A**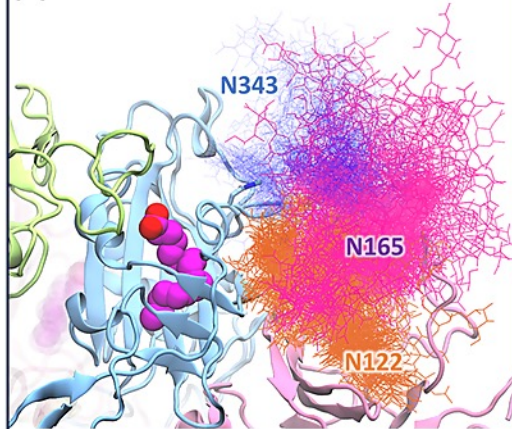**B****Path B**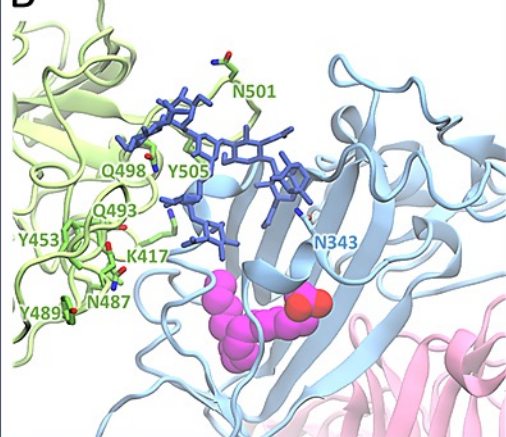**C****Path B**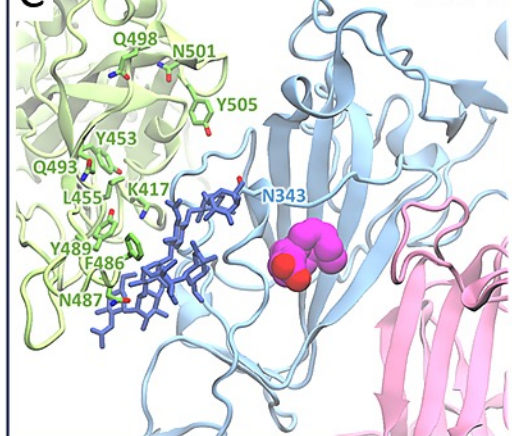**D****Path B**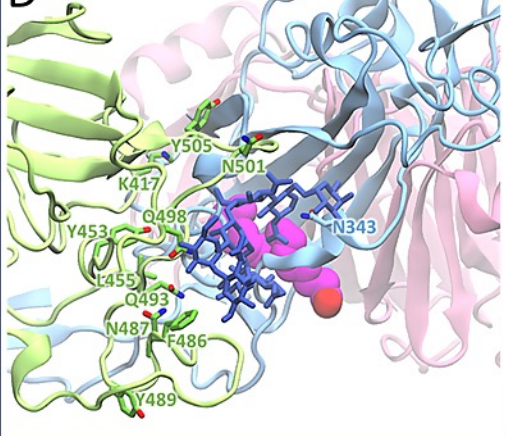

Supplement: S8 Fig — As LA dissociates from Chain A (blue) along Path A, the N343-glycan interacts with the glycans on N122 and N165 on the NTD in Chain B (pink) (A). Conversely, during LA dissociation along Path B, the glycan interacts with the residues of the RBM in Chain C (green), as illustrated in (B), (C), and (D). (PDF) [file pcbi.1011955.s016.pdf]
