# Supplementary material for: Mechanistic insights into ligand dissociation from the SARS-CoV-2 spike glycoprotein
Source: PLoS Comput Biol. 2024 Mar 7;20(3):e1011955. doi: 10.1371/journal.pcbi.1011955 (PMC10959368; doi:10.1371/journal.pcbi.1011955)
Supplement: S5 Fig — Line color represents RMSF profiles derived from trajectories during fully bound state (blue), traversal of Path A (green), traversal of Path B (orange), and after dissociation of the ligand (black). The highlighted colors correspond to different secondary structures: blue represents α1 (residues 337–343) and α3 (residues 364–369) helices, green corresponds to each β-strand (β2 residues 354–358, β3 residues 376–379, β5 residues 395–403, β6 residues 431–437, and β11 residues 507–514), and pink denotes the receptor-binding motif (RBM, residues 438 to 506). (PDF) [file pcbi.1011955.s013.pdf]

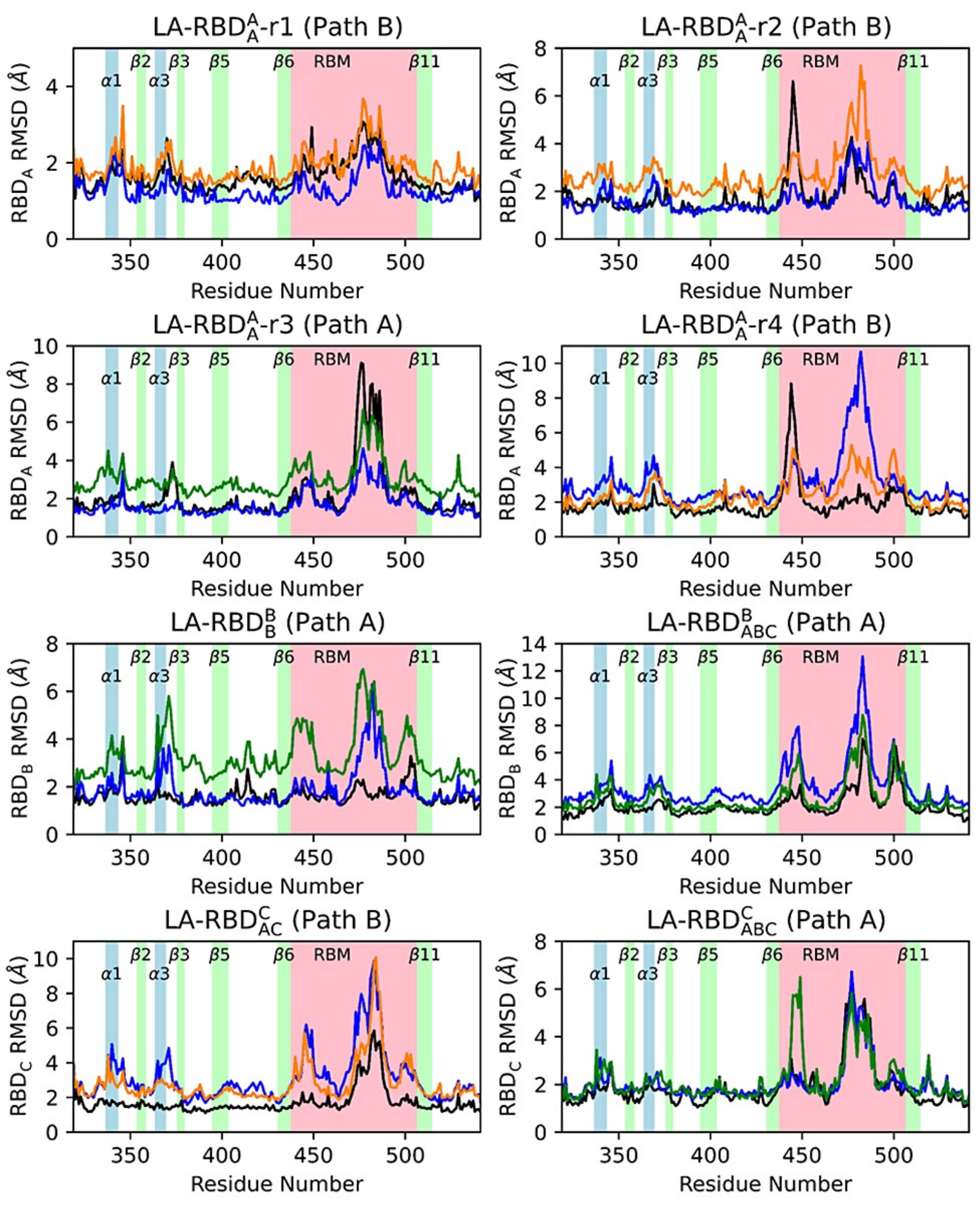

LA-RBD$^{A}_{A}$-r1 (Path B)
LA-RBD$^{A}_{A}$-r2 (Path B)
LA-RBD$^{A}_{A}$-r3 (Path A)
LA-RBD$^{A}_{A}$-r4 (Path B)
LA-RBD$^{B}_{B}$ (Path A)
LA-RBD$^{B}_{ABC}$ (Path A)
LA-RBD$^{C}_{AC}$ (Path B)
LA-RBD$^{C}_{ABC}$ (Path A)
$\alpha1$
$\alpha3$
$\beta2$
$\beta3$
$\beta5$
$\beta6$
RBM
$\beta11$
RBD$_A$ RMSD (Å)
RBD$_B$ RMSD (Å)
RBD$_C$ RMSD (Å)
Residue Number
