# Supplementary material for: Mechanistic insights into ligand dissociation from the SARS-CoV-2 spike glycoprotein
Source: PLoS Comput Biol. 2024 Mar 7;20(3):e1011955. doi: 10.1371/journal.pcbi.1011955 (PMC10959368; doi:10.1371/journal.pcbi.1011955)
Supplement: S6 Fig — It includes the open and closed distances as horizontal dashed lines, with the dissociation time indicated by a vertical dashed line. (PDF) [file pcbi.1011955.s014.pdf]

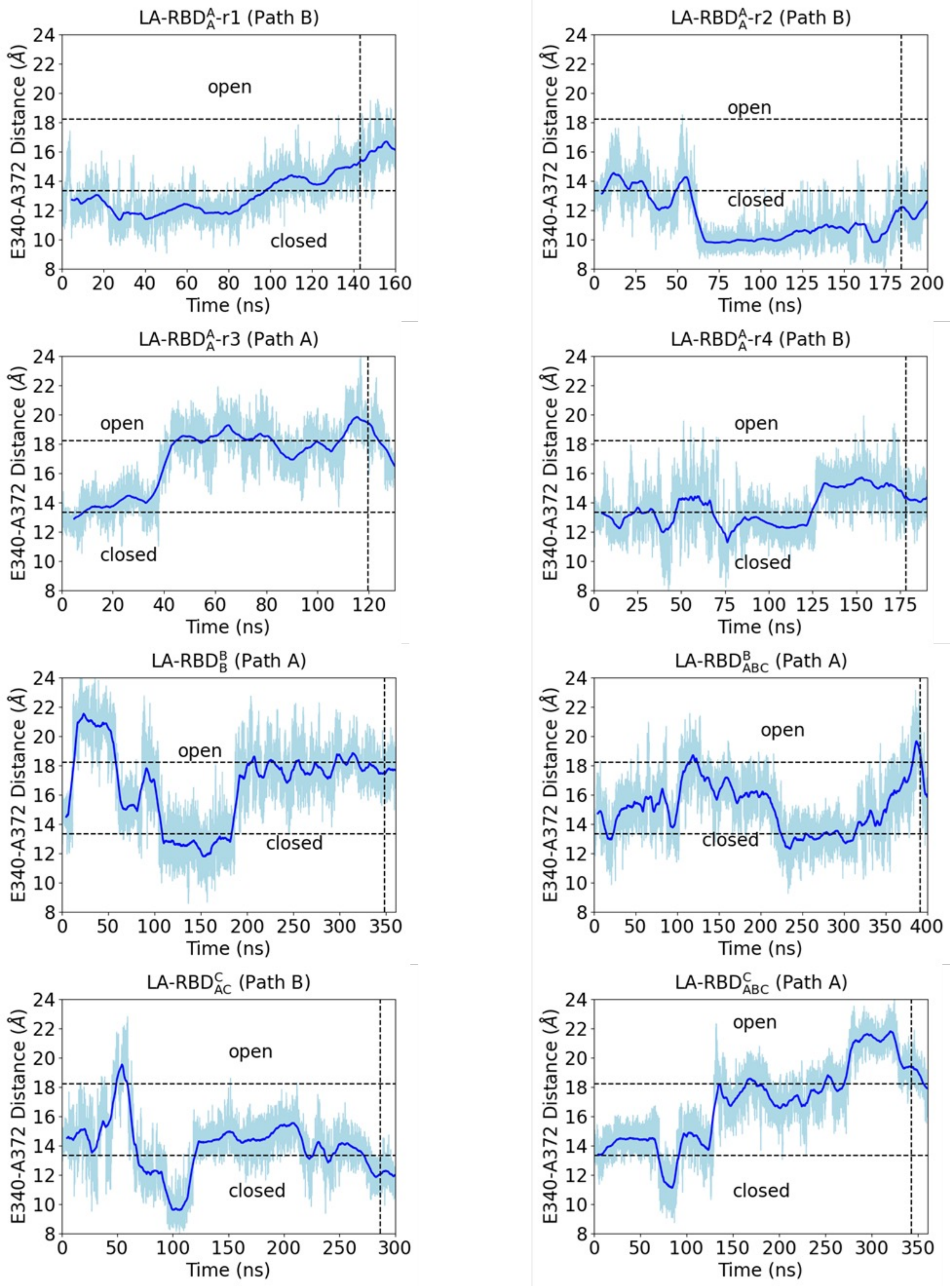

LA-RBD$^{A}_{A}$-r1 (Path B)
LA-RBD$^{A}_{A}$-r2 (Path B)
LA-RBD$^{A}_{A}$-r3 (Path A)
LA-RBD$^{A}_{A}$-r4 (Path B)
LA-RBD$^{B}_{B}$ (Path A)
LA-RBD$^{B}_{ABC}$ (Path A)
LA-RBD$^{C}_{AC}$ (Path B)
LA-RBD$^{C}_{ABC}$ (Path A)
E340-A372 Distance (Å)
Time (ns)
open
closed
